# Towards Automatic License Plate Detection

**DOI:** 10.3390/s22031245

**Published:** 2022-02-07

**Authors:** Zahid Mahmood, Khurram Khan, Uzair Khan, Syed Hasan Adil, Syed Saad Azhar Ali, Mohsin Shahzad

**Affiliations:** 1 Department of Electrical and Computer Engineering, Abbottabad Campus, COMSATS University Islamabad, Abbottabad 22060, Pakistan; zahid0987@cuiatd.edu.pk (Z.M.); uzairkhan@cuiatd.edu.pk (U.K.); 2 Department of Avionics Engineering, Air University, Islamabad 44000, Pakistan; khurram.jadoon@mail.au.edu.pk; 3 Faculty of Engineering, Sciences and Technology, Iqra University, Karachi 75500, Pakistan; hasan.adil@iqra.edu.pk; 4 Center for Intelligent Signal and Imaging Research (CISIR), Department of Electrical and Electronics Engineering, Universiti Teknologi PETRONAS, Seri Iskandar 32610, Malaysia; saad.azhar@utp.edu.my

**Keywords:** license plate detection, estimation, segmentation, object tracking, vehicle detection

## Abstract

Automatic License Plate Detection (ALPD) is an integral component of using computer vision approaches in Intelligent Transportation Systems (ITS). An accurate detection of vehicles’ license plates in images is a critical step that has a substantial impact on any ALPD system’s recognition rate. In this paper, we develop an efficient license plate detecting technique through the intelligent combination of Faster R-CNN along with digital image processing techniques. The proposed algorithm initially detects vehicle(s) in the input image through Faster R-CNN. Later, the located vehicle is analyzed by a robust License Plate Localization Module (LPLM). The LPLM module primarily uses color segmentation and processes the HSV image to detect the license plate in the input image. Moreover, the LPLM module employs morphological filtering and dimension analysis to find the license plate. Detailed trials on challenging PKU datasets demonstrate that the proposed method outperforms few recently developed methods by producing high license plates detection accuracy in much less execution time. The proposed work demonstrates a great feasibility for security and target detection applications.

## 1. Introduction

The ALPD is an important research field in the Intelligent Transportation Systems (ITS) domain. All vehicles in the world have license plates as their principal identifier. With the rapid development of machine vision technology, robust automated object detection methods are being introduced in the ITS [1]. An integral component of the ITS is vehicle License Plate Recognition (LPR), which identifies vehicles through character recognition on license plates [2,3]. Therefore, the LPD is the primary step in any LPR system because its detection accuracy and computational efficiency largely determines the overall performance of the whole system. Recently, a large number of LPD methods have appeared in literature. Many of them perform well in constrained environments, such as a single license plate in an input image with a simple background, fixed illumination, and a slightly distorted/blurred license plate [4]. Recent state-of-the-art techniques, such as [4,5,6], put less limits on object/license plate detection at the expense of higher computing complexity. Moreover, extracting license plates from complicated scenes remains a significant challenge for these methods.

Considering the aforementioned issues, this study presents a robust method to detect multiple license plates in different environments in near real-time. Furthermore, to further speed up the detection algorithm, we first use a fine-tuned F-RCNN to locate the vehicle position in the input image. Then, an LPLM is applied that uses basic image processing operations to locate the plate. Our proposed LPD method is motivated by common facts, such as license plates commonly have high edge density. In addition, characters on license plates are generally displayed in a horizontal orientation with nearly identical height. The major contributions of this study are as follows:

- We investigate challenging factors, such as very low or high luminance, bad lighting conditions, extreme reflective glare, and low-resolution on a challenging PKU dataset. In addition, we also introduce a novel mechanism that we refer as License Plate Localization Module (LPLM) in this manuscript. The proposed LPLM is efficient enough to handle challenging scenarios, such as extreme reflective glare, low, and high luminance variations. The LPLM works in near real-time and yields much higher license plate detection accuracy than few of the methods compared therein. To the best of our knowledge, illumination rectification has never been used in recently published works.
- We integrate the Faster R-CNN with our own developed License Plate Localization Module (LPLM), which emerges as a unique method to accurately detect different license plates that are provided by the PKU dataset developers. Moreover, the proposed method is significantly easier to use than previously reported methods, as it produces promising outcomes in difficult situations.
- Our proposed license plate detection algorithm outclasses several recent methods in terms of detection accuracy, precision, recall, and computational efficiency. Moreover, the proposed method is user-friendly and takes less than 3 s to detect the license plate in 1600 × 1200 pixels input image.

This paper is structured as follows. Section 2 briefly describes the recent developments in the license plates detection domain. The proposed method is elaborated in Section 3. Detailed simulation results are discussed in Section 4 along with the discussions and findings. Finally, Section 5, concludes the paper and hints towards the possible future directions. For the easy understandings of the readers, Table 1 lists the commonly used acronyms, symbols, and notations that are used extensively in this paper along with their meanings.

## 2. Related Work

As briefly reviewed in the following LPD approaches, research on LPD methods has made considerable progress in recent years for the effectiveness of any LPR system.

In [7], researchers’ developed a novel method to precisely locate and classify characters and license plate regions concurrently. In this work, an assembly layer is introduced for integrating the characters that outputs license plate strings. This method yields encouraging results on real world vehicles that contains license plates in different conditions. In [8], the problem of the LPD is tackled by pixels to pixels investigation. Researchers integrated the Adaboost, which is an intelligent classifier with the SIFT-based SVM classifier. In [9], the HOG-based features of a segmented license plate are fed to the Adaboost classifier to locate license plates. In [10], the developed methodology addresses challenges in license plate detection mainly through color transformations and variations in various color spaces through segmentation and filtering procedures. In [11], the proposed approach has three steps to locate a car license plate. Initially, a Sliding Concentric Windows (SCWs)-based methodology is introduced to extract candidate regions, followed by the HSI-color model to identify those candidate regions. In the final stage, the candidate regions are decomposed using a position histogram to figure out characters in the plate. Kim et al. [12] integrated color and texture features to locate complex license plates, whereas [13] proposed a fixed color-pairs-based license plate localization method to process the plate characters and background regions. In [14], a modified template-matching algorithm is presented through color pixel analysis. In [15], a fully Convolutional Neural Network (CNN) was deployed to extract features at different stages of the CNN to differentiate the details of the plates and the background followed by a three loss layer architecture for accurate plate detection.

In [16], a YOLO V3 tiny object detector is introduced to detect license plates on images of the high efficiency video coding domain. This work reports a new compressed domain license plate database, which comprises images that are captured by a commercial license plate recognition system. Using at least two-orders-of-magnitude less amount of data, this work reports higher detection results than few of the methods compared therein. Furthermore [17], addressed the license plate detection problem using a Deep Convolutional Neural Network (DCNN) and Long Short-Term Memory (LSTM) in natural scene images. In [18], the work discusses a method to locate Chinese license plates using color detection and segmentation. In [19], a method uses the Faster-RCNN integrated with a hierarchical sampling method to detect the license plate. In [20], license plate detection is achieved through deep learning and character segmentation. In [21], the developed license plate detection technique applies image downscaling and a line density filter approach. In [22], the researchers integrated the component-based approach with a conditional random field model to locate the license plates. In [23], a detection technique for rotated license plates based on the CNN is proposed. Meanwhile, [24] used the concept of principal visual word using the bag of words to locate license plates in various environments. A fully convolutional neural-network-based approach [25] handles the problem of object detection through shared computations on the whole image. Finally, a recent advancement in the license plate domain can be seen in [26], where authors propose an intelligent license plate detection and recognition system through intelligent utilization of neural networks and a hybrid-based broad learning system.

The methods detailed above are just a few of the many that have been published in the field of license plate detection. However, due to issues, such as the comparably tiny areas of license plates, varied backdrop clutters, many plates being in an image, and non-uniform illuminations, robust and reliable license plate detection remains a difficult task. Therefore, our developed method is one of the newest additions in the research domain that aims to locate license plates in complex scenarios, such as low or high luminance, reflective glare, and multiple license plates per image on a challenging PKU dataset as described in the next section.

## 3. Proposed Method

To achieve the reliable, fast, and accurate vehicle license plate localization, the proposed method has two major modules:

- **Vehicle detection:** Our motivation to detect vehicles comes from the rising technology in the computer vision field, which has recently reported great success in computer vision tasks, such as object detection and classification. Therefore, computer vision provides a significant improvement in data volume and rapid hardware advancement. At present, there are various object detection algorithms with excellent real-time performance. In our work, to detect an object, such as vehicle(s), a Faster R-CNN is used as detailed in the next section.
- **License plate detection:** It is achieved through authors’ proposed License Plate Localization Module (LPLM). As we will describe in Section 3.2, the proposed LPLM works reliably under diverse conditions.

**Figure 1:**
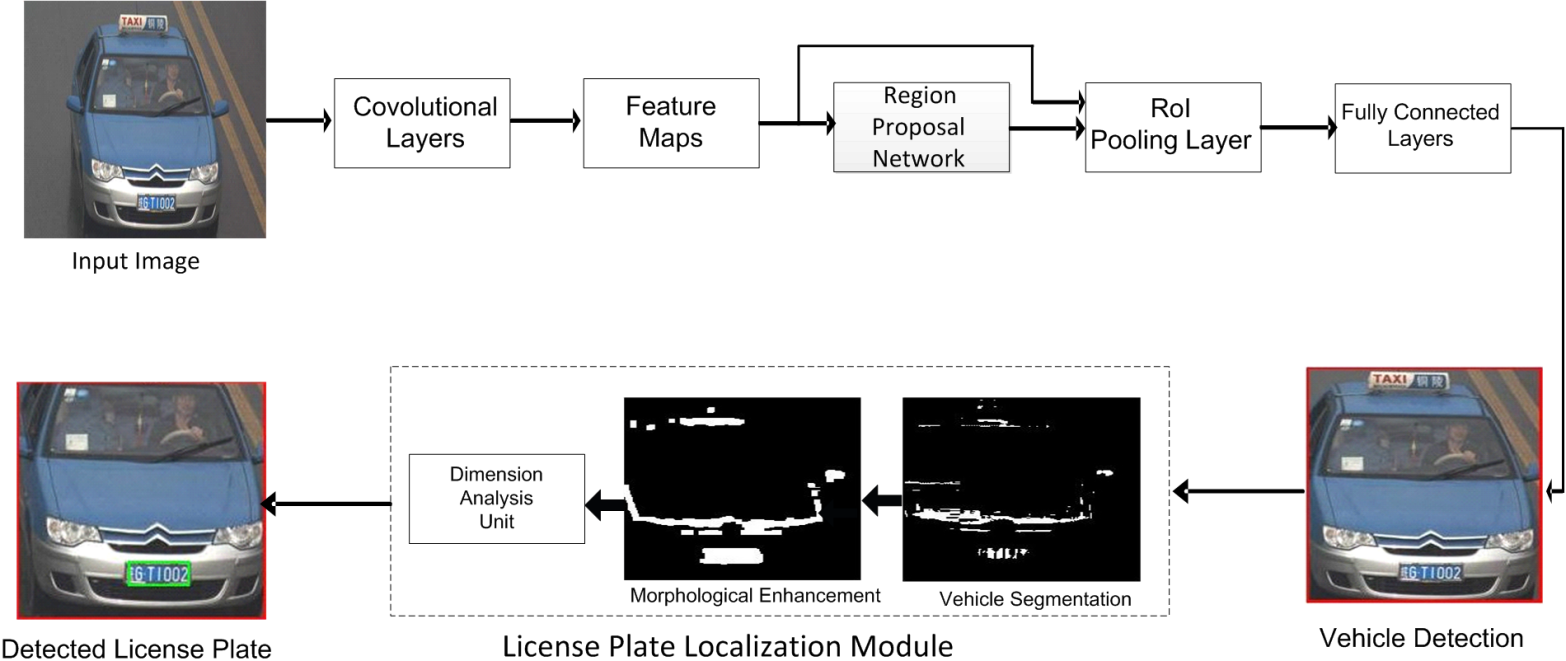
Block diagram of proposed method.

Figure 1 shows the complete steps of developed license plate detection algorithm. The proposed method has various interconnected modules. In the sections below, we briefly describe these modules.

### 3.1. Vehicle Detection

The aim to detect vehicles is to consider the accurate and fast detection of vehicles in the PKU dataset images. Two critical conditions should be satisfied by any vehicle detector, which are (**i**) near real-time detection and (**ii**) high detection accuracy of the traffic objects. During the past two decades, various challenging vehicle detection benchmarks have been introduced for evaluation of detection algorithms. Among them, deep-learning-based methods have achieved impressive achievements on vehicle detection. In general, R-CNN, Fast R-CNN, and Faster R-CNN are the recent methods that have high accuracy, but have huge computational complexity and are difficult to meet real-time performance. To effectively and quickly detect a license plate, we initially spot the vehicle in the image through Faster R-CNN [27]. The purpose of detecting the vehicle is to limit the area that has to be searched for the license plate in the next stage. The intention to introduce Faster R-CNN in initial stage is that it is at least nine times quicker than the R-CNN during the training stage. Moreover, it is 213 times faster at test time with much higher detection accuracy [27].

Algorithm 1 shows the pseudo code of our developed algorithm for license plate detection in which the vehicle detection module is applied from lines (1) to (21). From lines (3)–(10), we perform the fine tuning of the Faster R-CNN classifier to extract 64 RoIs from the input image. To label the foreground object mask, we select an object proposal with an Intersection over Union (IoU) overlap with the ground truth of at least 0.5. From lines (11)–(12), a colored RGB vehicle image is processed with 13 convolutional (conv) layers to obtain a conv feature map (ψ). Then, for each proposed region of the vehicle Region Proposal Network (RPN), nine different anchors are applied on ψ, which predicts the potential vehicle candidate regions. It is important to state here that for anchors, three scales are used with box areas of $128^{2}$, $256^{2}$, and $512^{2}$ pixels, and three aspect ratios of 1:1, 1:2, and 2:1. In line (15), we perform max pooling using five layers on $\left(\frac{h}{Height}\times \frac{w}{Width}\right)$ with a 7×7 of Height×Width with *h* and *w* as the layer hyper-parameters. For readers’ information, it is important to state that these parameters are independent of any particular RoI.
**Algorithm 1:** Proposed license plate detection procedure.
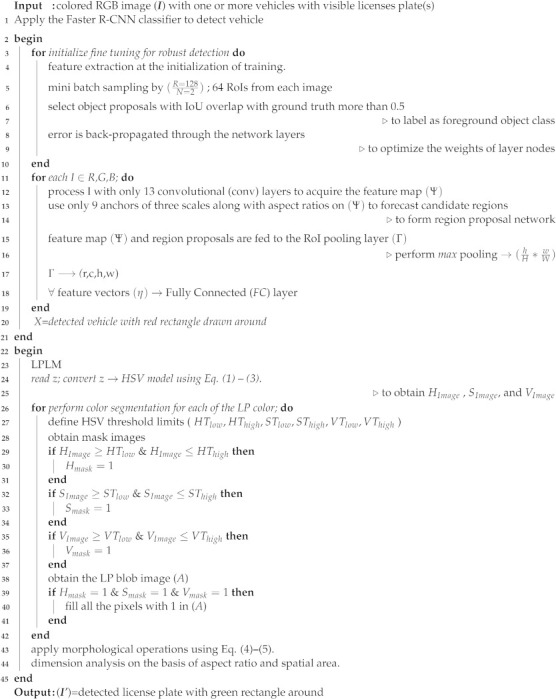


The RoI is viewed in this work as a rectangular window onto (ψ). Furthermore, each RoI is represented by a four-tuple (r,c,h,w) that specifies its top-left corner (r,c) and its bottom-right corner (h,w) (h,w). Every feature vector (Γ) is fed into a series of Fully Connected (FC) layers as an input. The RoI pooling layer, on the other hand, uses max-pooling to turn the features inside a binding zone into a compact feature map. Furthermore, only a few RPN concepts have a lot of overlap. As a result, we use Non-Maximum Suppression (NMS) [27] on the proposal regions to eliminate redundancy. For NMS≥0.5, we set the IoU threshold to 2000 proposal regions per picture, resulting in a considerable reduction in the amount of suggestions. Following the NMS, the top-N rated proposal regions are predicted to detect the vehicle in line (20) of Algorithm 1 by drawing a red rectangle around the vehicle.

Once the vehicle(s) are spotted in the input image, in next step, we use our own developed LPLM methodology to detect the license plate within the detected vehicle image. In the next section, we describe our developed LPLM that takes the vehicle detected image as input.

### 3.2. The License Plate Localization Module (LPLM)

The processed image that contains the spotted vehicle (*X*), which is obtained in line (20) of Algorithm 1, is fed to the LPLM. The LPLM after processing the vehicle detected image locates the position of license plate(s) within the detected vehicle region. The proposed LPLM module has three major interconnected steps, which are (**a**) RGB to HSV domain transformation, (**b**) Morphological operations application, and (**c**) Dimensions analysis. Below, we briefly define these modules.

#### 3.2.1. RGB to HSV Transformation

The LPLM initially processes the RGB image and transforms it into the HSV domain, as shown in Equations (1)–(3):

Max=max(R,G,B)andMin=min(R,G,B)

(1) $$H=\left\{\begin{array}{cc} 0 & ifMax=Min\\ \;\;60\times \frac{G-B}{Max-Min}+0 & ifMax=R,G\geq R\\ 60\times \frac{B-R}{Max-Min}+120 & ifMax=G\\ 60\times \frac{R-G}{Max-Min}+240 & ifMax=B\\ 60\times \frac{G-B}{Max-Min}+360 & ifMax=R,G<B \end{array}\right.$$

(2) $$S=\left\{\begin{array}{cc} \;0 & ifMax=0\\ \;\frac{Max-Min}{Min} & otherwise \end{array}\right.$$

(3) V=Max

where RGB denotes original Red, Green, and Blue components, respectively. The license plate in the PKU dataset has a diverse variety of colors. Therefore, we apply the color segmentation from lines (26)–(38) of Algorithm 1 on each of the HSV components to obtain the LP blob image (*A*) as shown in line (38) of Algorithm 1. As the RGB image is transformed to the HSV image, we obtain three channels that we refer to as H*Image*, S*Image*, and V*Image* in line (25) of Algorithm 1. In line (26), color segmentation is applied on each of the HSV image channels that were obtained in the above step. For each of the HSV channels, a low Threshold (T*low*) and a high Threshold (T*high*) are defined. For each of the HSV channels, a range of low and high Thresholds are applied. As a result, for the Hue channel, its relevant low and high Thresholds are HT*low* and HT*high*, respectively. During our simulations, we vary the HT*low* value from 0.02–0.40 and HT*high* from 0.40 to 0.62. Similarly, for the Saturation and Value channels, their relevant low and high Thresholds are ST*low*, ST*high*, VT*low*, and VT*high*, respectively, as shown in line (32)–(35) of Algorithm 1. For ST*low*, the values are varied from 0.37–0.50, whereas for ST*high*, they are varied from 0.90 to 1.0. For the V channel in the HSV image, the VT*low* is set to 0.75 and the VT*high* is set to 1.0. After the Thresholds are set, now the mask images are obtained for each of the H, S, and V channels as shown in lines (30)–(36) of Algorithm 1. For the H channel, the H*mask* is set to 1 when the H*Image* obtained in line (25) of Algorithm 1 is greater than or equal to the low Threshold and less than or equal to the high Threshold. A similar mechanism is applied to obtain the masks of the S and V channels, as shown in lines (33)–(36) of Algorithm 1. Finally, a license plate’s blob image (*A*) is obtained as shown in line (38). This blob image is filled with binary value 1 if, and only if, the values in the H*mask*, S*mask*, and V*mask* are all equal to 1 through logical AND operation. Otherwise, values in blob image are set to value 0. After the segmentation is complete, in the next step, morphological operations are applied as described below.

#### 3.2.2. Morphological Operations

In order to boost the license plates blobs in the sample space (*z*), the below-mentioned morphological operations are applied to the obtained blob image (*A*).

**Dilation** (⊕) is performed using Equation (4) that expands the features by inserting a layer of pixels around the regions of the linked components. As a result, the obtained license plate blob is expanded:

(4) $$A⊕B=\{z|\left(B^{\wedge }\right)z\cap A\neq \phi \}$$

where *B* denotes the structuring element by which A is dilated. Dilation is followed by the Closing operation as discussed below.

**Closing** (·) is performed using Equation (5). The blob image *A* is initially processed by dilation operation and later eroded by a structuring element *B*, which results in the contour smoothing and holes filling in the license plate blob:

(5) A·B=(A⊕B)⊖B

When the luminance is too low or high, as is the case in the PKU-G5 category with extreme reflective glare, we propose another methodology, as shown in Algorithm 2. To handle the aforementioned scenario, we apply the PCA [28] from lines (1)–(8) in Algorithm 2 on the detected input vehicle image. The application of the PCA results in the extraction of the luminance and chrominance channels. In our method, to reduce our algorithm’s computational cost, we only process the luminance channel in later steps. After the PCA is applied, in next step, we calculate the mean of the luminance vector as shown in line (9) of the Algorithm 2. Meanwhile, we empirically estimate the low and upper limits of threshold as shown in line (10) in Algorithm 2. In our simulations, we set the value of threshold*low* to 0.25 and threshold*high* to 0.95. From lines (11)–(13), the luminance is adjusted to obtain the final neat and clean enhanced output image (X’). The enhanced output image with much better and stable luminance is later processed by the License Plate Localization Module (LPLM).
**Algorithm 2: **Pseudo code to handle the reflective glare scenario.
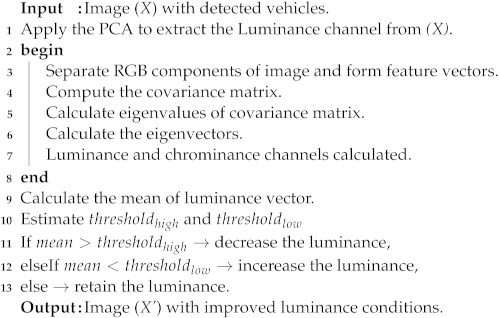


#### 3.2.3. Dimensions Analysis

Once the Connected Regions (CR) are generated and enhanced by morphological operations, we examine the dimensions of the extracted regions to locate the license plate. We calculate the dimensional parameters of the CRs and examine whether its values fall in the range of the targeted license plate dimensions by using the two following parameters:

- **The Spatial Area (SA)**, which is a collection of pixels in a spatial region that are confined within bounding box. In this study, a premeditated SA of the CRs is compared to the learned SA of the desired license plate.
- **The Aspect Ratio (AR)**, which is defined as the relation of height-to-width in a license plate. After the SA confirmation, if the AR parameter equates with the learned license plate parameters, it endorses the existence of the license plates. Finally, the LPLM module draws the green rectangle on that connected region, which outlines the existed license plate in the input image.

## 4. Results and Discussion

We use Dell Precision Tower 7810, Dual Intel Xeon Processor E5-2699 V3 machine that has 192 GB of RAM. We execute all the simulations in C++. We compare our developed method with [21,22,23,24,25] in terms of precision, recall, overall detection accuracy, and execution time on the PKU dataset [21] as described in Table 2.

### 4.1. Vehicle Detection Accuracy

**Figure 2:**
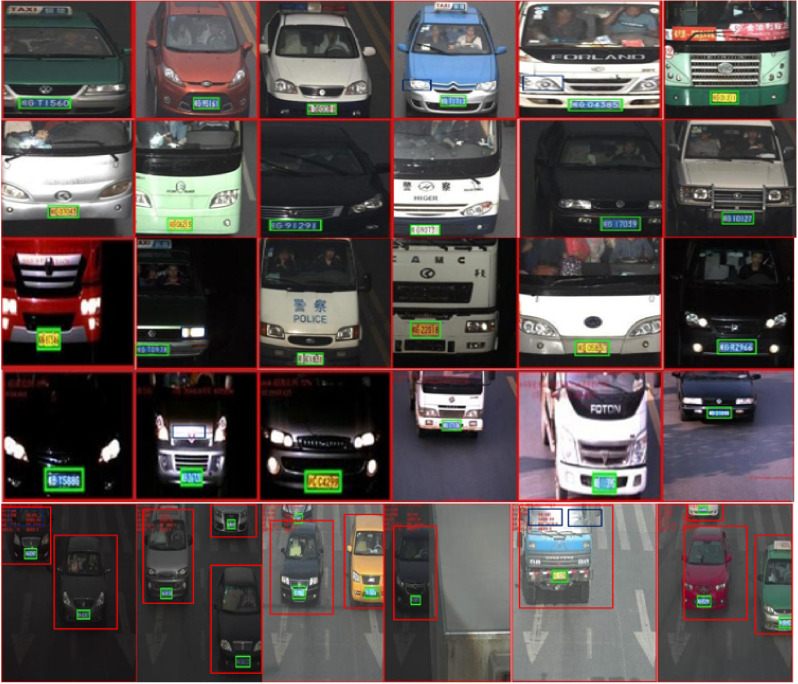
Detection results: first row: G1; second row: G2; third row: G3; fourth row: G4; and fifth row: G5.

Since the first step in the localization of license plates is to detect the position of the vehicle, we therefore apply the Faster R-CNN in the initial stage to understand the feasibility of the vehicle detection module. Figure 2 shows a few of the images with the detected vehicles by our proposed methodology. From Figure 2, a few important observations are described below:

- As shown in top three rows of Figure 2, the vehicle detection module is intelligent. It detects all the vehicles that have the diversity in appearance. We observe that the vehicle detection module has 100% detection accuracy on G1, G2, and G3 categories. The input vehicle image resolution in these categories varies from 420 × 280 up to 600 × 340 pixels. Similarly, the bottom row in Figure 2 shows 13 vehicles/license plates with 100% detection result.
- Similarly, in the fourth row of Figure 2, it is quite evident that the vehicle detection module accurately locates the partially appeared vehicle in the input image. Moreover, a few of the vehicles in the fourth row have their head lights on, which generates the phenomenon of non-uniform illuminations. However, the detection module precisely detects all vehicles in the first four rows of Figure 2. The vehicle detection module has 99.7% detection accuracy on the G4 category, which has the input image resolution of 400 × 320 pixels.
- Images shown in the fifth row of the Figure 2 are taken from G5 category, which has multiple vehicles under different test conditions. However, we see that the vehicle detection module locates all vehicles. On the PKU G5 category with input vehicle resolution of 300 × 270 pixels, the vehicle detector module yields 99.1% detection accuracy.

Table 3 summaries the vehicle detection results. From Table 3, important findings are listed below:

- As can be seen in Table 3, the vehicle detection module is robust to detect diverse vehicles in the PKU dataset. For the G1–G3 categories, the vehicle detection module detects all the vehicles in the PKU dataset.
- It is evident from Table 3 that the vehicle detection module is robust to detect diverse vehicles in the PKU dataset. For the most challenging PKU-G5 category, the vehicle detection module yields over 99% detection accuracy. As shown in Table 3, the vehicle detection module yields an average detection accuracy of 99.76% on the PKU dataset. This result is encouraging, as many of the vehicles in the PKU dataset are partially occluded. In addition, a large number of vehicle images become more challenging as the vehicles therein just expose the bonnet as shown in the bottom row of Figure 2. Therefore, the vehicle detection module accurately handles the aforementioned scenarios.

### 4.2. License Plate Localization Module (LPLM) Accuracy

After the LPLM is applied, we observe that each license plate in the PKU dataset is contained by a green color bounding box. Therefore, we evaluate the license plate detection performance of the LPLM in terms of the localization accuracy. The discussion below mostly focuses on the accuracy of the LPLM module:

- Figure 2 shows the detection results of the G1–G5 categories. As shown in Figure 2, the proposed LPD algorithm accurately detects the license plates for diverse vehicle shapes and colors. In particular, all six license plates in the top row of Figure 2 are located by the LPLM.
- Moreover, for severe dark contrast, as shown in the second and third rows of Figure 2, all the license plates have been detected successfully. This case is also interesting as almost all of the images shown in Figure 2 have entirely different frontal appearances.
- For the G4 category, as shown in the fourth row of Figure 2, the proposed LPD algorithm effectively handles various license plates. The proposed method is robust as it is not affected by the variations in contrast and illuminations.
- The G5 category in the PKU dataset contains a huge variety of vehicles, such as plates with multiple orientations and vehicles diversity. However, as seen in the bottom row of Figure 2, the proposed algorithm accurately handles the aforementioned issues of the G5 category.

**Figure 3:**
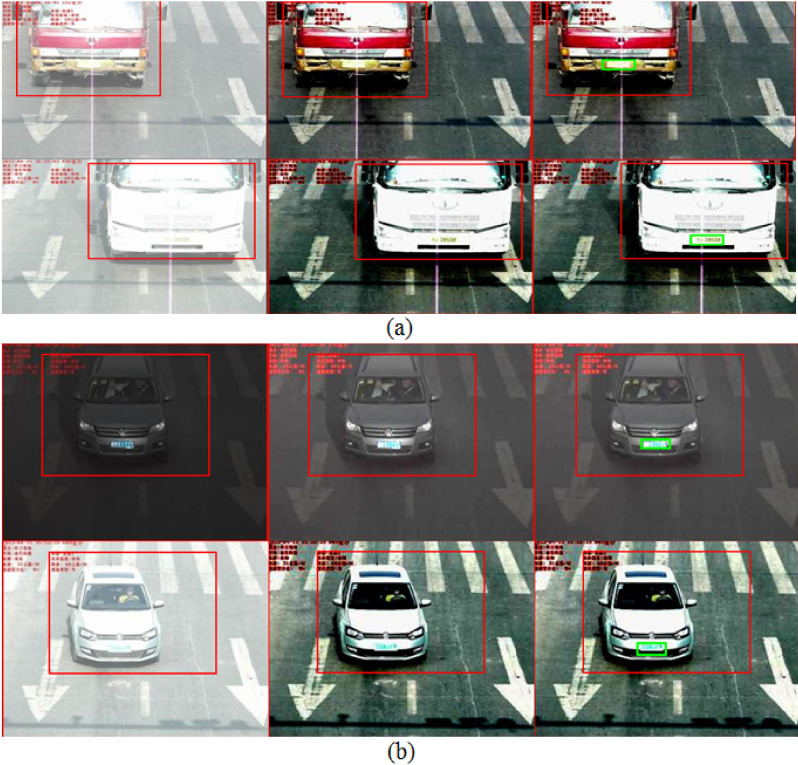
Detection results on (**a**) PKU-G5 extreme reflective glare and (**b**) top-row: low luminance and bottom-row: excessive luminance.

Table 4 summarizes the results for each of the category on the PKU dataset. From Table 4, a few of the important findings and observations are discussed below:

- On the PKU G1–G4 categories, the proposed license plate detection method outperforms [21,22,23]. It is important to state here that in G1 to G4 categories the input images contain only one license plate per image under different appearances. For instance, each of these categories contain images of cars and trucks that have been captured at different times of day and night and in different sun shines. The G4 category in the PKU dataset is a slightly challenging category as out of 572 images, it contains 252 reflective glare images. However, the proposed method is efficient enough and beats all of the compared methods in terms of detection accuracy.
- The G5 category in the PKU dataset is most challenging among all because it contains several vehicles and thus multiple license plates per image. Moreover, the G5 category also contains roads junctions as well crosswalks. Furthermore, it also has low and excessive luminance phenomenon, which makes it a challenging scenario for any machine learning algorithm. As seen from Table 4, in the G5 category, the proposed method ranks third among the compared methods. In this category, [23] yields the highest detection accuracy of 97.38%, whereas [21] reports 97.32% detection accuracy.
- As discussed in Section 4.6 and also shown in Figure 3a, non-uniform illuminations present a major threat to the detection of a license plate in unconstrained environments. Therefore, due to the aforementioned problem, works in [24,25] report relatively low detection accuracies of 82.37% and 83.81%, respectively. Quantitatively, the proposed method on average achieves the highest license plate detection rate of 99.04% on the whole PKU dataset (G1–G5), followed by [23], whose detection accuracy is reported to be 97.38%. The work presented in [25] reports the least accuracy of 83.81% on the PKU dataset.
- Our findings suggest that illumination variation poses a major threat to any object detection and recognition algorithm, such as face detection, pedestrian detection, face recognition, and even a license plate recognition system. A license plate recognition system is highly dependent upon accurate and precise features being fed by the detector module. Therefore, we feel it is suitable to investigate the non-uniform illumination variations in the PKU G5 category.
- To further test the robustness of the LPLM, we manually reduced the input image resolution up to 320 × 240 pixels. On the aforesaid image resolution, the LPLM accurately detected the license plate that had the resolution of 28 × 19 pixels.

### 4.3. Evaluation on Extreme Reflective Glare, Low, and High Luminance

To further evaluate the robustness of proposed method as described in Algorithm 2, we test our algorithm on extreme reflective glare conditions as described in Figure 3a,b. In this study, we investigate three challenges. First is the extreme reflective glare scenario, as shown in Figure 3a. The second challenge is low-luminance, as depicted in top row of Figure 3b, and the final challenge is the excessive luminance condition as shown in bottom row of Figure 3b. For each of these challenges, our important observations are highlighted below:

- For the extreme reflective glare scenario, as shown in top row of Figure 3a, only the vehicle is detected but not the license plate. However, after the proposed reflective glare mechanism is applied, it is evident in the third image of the top row in Figure 3a that the license plate is accurately detected. Moreover, the quality of the image is now improved with a much more pleasant outlook. Similarly, the second row in Figure 3a is even more challenging phenomenon in which the color of the vehicle is also white along with the reflective glare. In such conditions, only the vehicle is detected. After we apply the glare rectification mechanism, it can be seen in second and third images of Figure 3a that the image quality has improved along with the accurate detection of the license plate.
- For the low-luminance scenario, as shown in the top row of Figure 3b, only the vehicle is detected. However, after the proposed luminance rectification method is applied, it is evident in the third image of the top row in Figure 3b that the license plate is now precisely detected.
- For the excessive-luminance scenario, as shown in second row of Figure 3b, only the vehicle is detected. In this image, the bonnet and the license plate area are barely differentiable through naked eye. As can be seen after the proposed luminance rectification method is applied, the license plate is exactly detected.
- To the best of our knowledge, none of the previous works [21,22,23,24,25] address reflective glare scenarios. It is important to state here that the vehicle detection module is robust and precisely locates vehicles under severe dark or over bright contrast. The images shown in Figure 3b depict the bad lighting conditions scenario. However, the proposed method handles the aforesaid scenario efficiently.
- It is important to state here that such a reflective scenario is found only in the G4 and G5 categories. Out of the 572 test images from the G4 category, we found and analyzed 252 test images to be affected by the reflective glare. Meanwhile, from the 1152 test images of the G5 category, we found 706 images to be either low/high luminance and affected by extreme reflective glare. In our simulations, we have tested all such images, and our findings are summarized in Table 4. Due to these variations, the published works in [22,24,25] report much lower detection accuracy in the G5 category. The works in [21,23] report slightly higher detection accuracy in the G5 category than the proposed method. However, the proposed method outperforms all works published in [21,22,23,24,25] in terms of the mean accuracy on all five categories of the PKU vehicle license plate dataset.

### 4.4. Precision and Recall

We empirically compute the precision as well as the recall parameters of the compared techniques of several intervals of matching confidence (ξ). The aforesaid analysis is performed between the forecasted rectangle drawn by our method (*c*) along with its ground truth (*t*). It is worthwhile to describe here that the matching confidence (ξ) is calculated using Equation (6):

(6) ξ={c∩t/c∪t|t⊆c}

**Figure 4:**
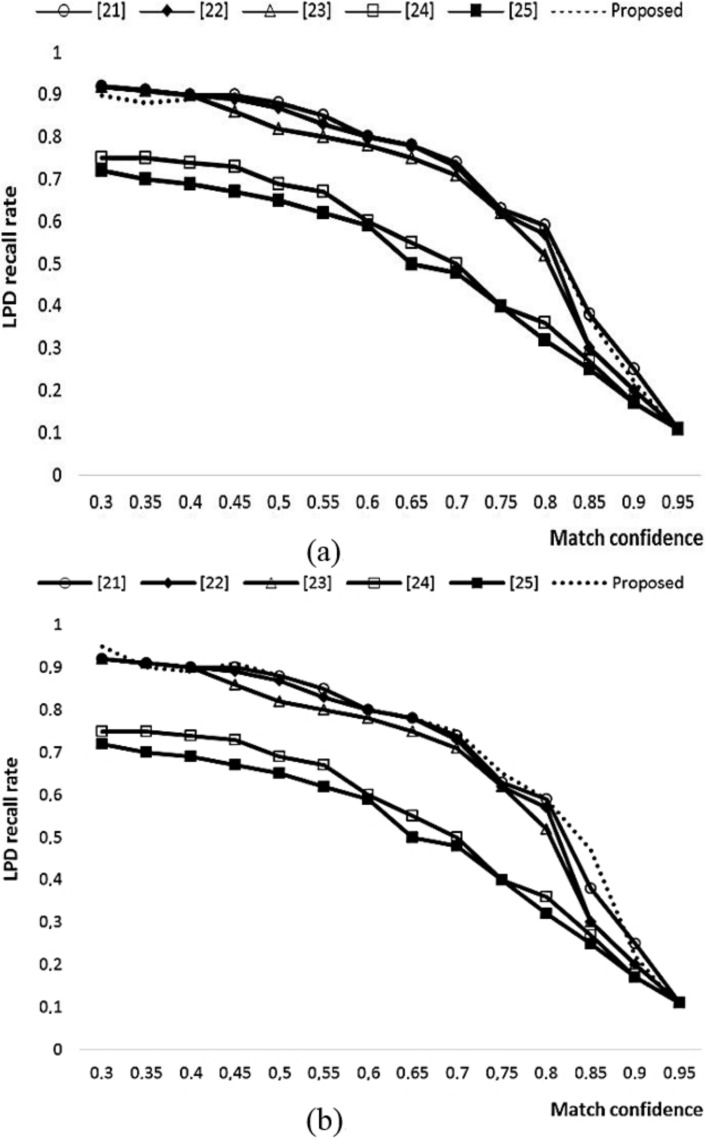
(**a**) Precision and (**b**) recall comparison.

For matching confidence ($\xi =\xi_{0}$), both the *Precision* and *Recall* are calculated using Equations (7) and (8), respectively:

(7) $$Precision=\frac{TP}{TP+FP}$$

(8) $$\begin{array}{c} Recall=\frac{TP}{TP+FN} \end{array}$$

where *TP* denotes True Positives and implies the actual detection of license plate(s). The higher the *TP* rate is, the higher the *Precision* and *Recall* ratio will be. Few examples of *TP* are actual detected license plates, which are enclosed by green rectangles as shown in Figure 2. The *FP* represents False positives, which is the case of a non-license plate region being detected as a license plate by the detector. A higher value of *FP* will result in a low *Precision* ratio. One such example of *FP* is found in the fifth image of the last row of Figure 2, where two blue rectangles on the front mirror of a truck are detected as a license plate area by the detector. *FN* represents False Negatives. A high value of *FN* will result in low *Recall* ratio. Few examples of *FN* are shown in the first two columns of Figure 3a,b where the actual license plates present in the image are not located by the detector due to reflective glare and luminance variations. A good algorithm should have higher values of both *Precision* and *Recall*. The comparison of the *Precision* and *Recall* values for different matching confidence intervals is given in Figure 4a,b. As can be seen in Figure 4a, for the matching intervals from 0.33 to 0.41 and greater than 0.84, the methods in [21,22] have slightly higher *Precision* than the proposed method. For all other interval range, the proposed method has comparable *Precision* values with the compared methods. Moreover, in Figure 4b, it is clear that the proposed algorithm yields higher *Recall* values when the range of matching confidence interval exceeds 0.40.

### 4.5. Image Variations Analysis

**Figure 5:**
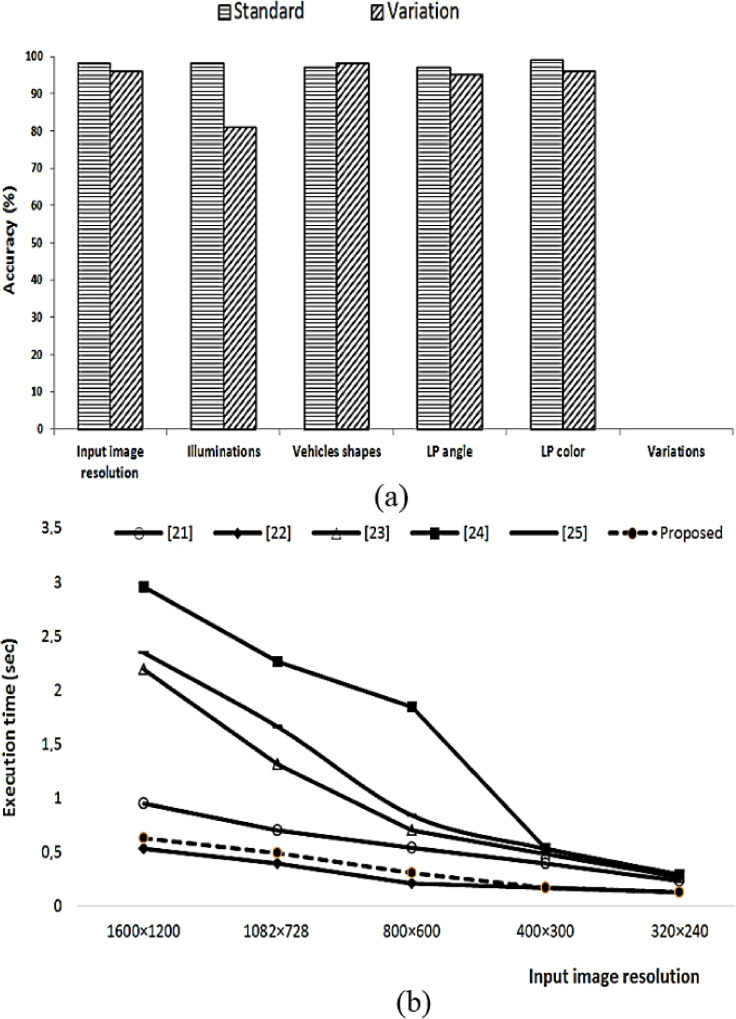
(**a**) Variation in parameters and (**b**) Computational complexity comparison.

To validate the robustness of our developed LPD algorithm, in Figure 5a we show the license plate detection accuracy for five important variations, which are **(i)** image resolution, (**ii**) illumination variation, (**iii**) vehicle shapes, (**iv**) license plate angle, and (**v**) license plate color. For each of the variations, as shown in Figure 5a, there are two towers. The first tower depicts the standard conditions as provided by the PKU dataset, while the second tower shows the performance of the proposed algorithm when the relevant parameter is varied. From Figure 5a, a few important observations are in order:

- We examine the five different image resolutions, which are 1600 × 1200, 1082 × 728, 800 × 600, 400 × 300, and 320 × 240 pixels image resolutions. As shown in first pair of towers in Figure 5a, the detection accuracy of the proposed algorithm has barely any effect on performance with the variation in image resolution. In our simulations, we observe that as the image resolution is decreased from 300 × 240 pixels, the detection performance of the proposed algorithm slightly starts to degrade.
- As shown in the second pair of towers in Figure 5a, the detection accuracy of the proposed algorithm has a minor effect with the variations in illumination conditions. In the PKU dataset’s illumination variations, our proposed algorithm yields 97.30% detection accuracy. However, when we manually varied the contrast of the input images, the accuracy of the proposed algorithm dropped down to 82.78%.
- Our detailed analysis for different shapes of the vehicles outside the PKU dataset reveals that the vehicle detection module is also robust and virtually detects every available vehicle shape.
- In the PKU dataset, the provided license plates have yellow, blue, and white colors. We also tested the performance of our developed algorithm for green and light/dark blue colors. As shown in the fifth pair in Figure 5a, license plate colors variation have the least effect on the detection accuracy of the proposed algorithm. Similarly, the license plate rotation or angle variation has a minor effect on the performance of proposed algorithm. In all of the previously described cases, the license plate detection accuracy is over 95% for the parameters outside the PKU dataset.

### 4.6. Computational Complexity

The proposed method is compared with recent state-of-the-art methods in terms of average run times in Figure 5b for various image resolutions. From Figure 5b, it is obvious that the proposed approach ranks second and requires only 0.551 s to detect a license plate for input image resolution of 1600 × 1200 pixels. Zhou’s [24] approach is most computationally expensive, and for an input image resolution of 1600 × 1200 pixels, it requires approximately 3 s to locate a license plate. Therefore, we believe that the proposed approach is robust and efficient. The component-based approach [22] is the most computationally economical in our study, and for large image resolution, such as 1600 × 1200 pixels, it takes about 0.487 s to accomplish the detection task.

### 4.7. Qualitative Analysis

Although Figure 2 and Figure 3 show some examples of license plate detection by the proposed approach on the PKU dataset, the points mentioned below highlight some important observations during our simulations:

- The license plates were located accurately in the images with: (**a**) a normal environment having a single license plate and (**b**) slight sunshine at different day times, as is the case in the PKU-G1–G2 categories. A few of such images are shown in Figure 2. However, it also extracts irrelevant candidate regions (false positives), which are indicated by blue rectangles in the fifth row in Figure 2.
- Images in the PKU-G3 category are captured mostly at night, where the light conditions are very poor with low illuminations. The third row in Figure 2 reveals that the proposed technique handles these conditions effectively. Similarly, in the PKU-G4 category, approximately half of the images are captured at night, and in most images, only the bonnets and bumpers of the vehicles are visible. The fourth row in Figure 2 shows that the proposed LPD algorithm accurately works under the aforementioned conditions.
- The PKU-G5 category is the most challenging category in the dataset. Most of the images have huge variations in the illuminations with multiple vehicles and complex backgrounds, as shown in Figure 3. In this category, we observe that our proposed method generates additional candidate regions mostly around the red text in the PKU database and near the region of high beam intensity. However, Figure 3 also reveals that our proposed algorithm is able to accurately locate the exact positions of the license plates. For complex images/video frames, a suitable complexity reduction method might be needed before license plate localization [9,10].

### 4.8. Discussion

Although Section 4 sheds detailed light on the validity of the proposed algorithm points. However, discussion below gives further insight about the feasibility of the proposed algorithm.

- The aforementioned qualitative analysis demonstrates that our proposed license plate detection algorithm outperforms the previous published methods. We observe that such improved performance has been obtained due to precise development of LPLM as described in Algorithms 1 and 2, which exactly handles the image variations as shown in Figure 5a.
- Although our method handles the license plate angle variation as shown in the PKU dataset, we observe that in the PKU dataset the LP angle variation is just the deviation of the image capturing device that results in the view variation. However, to accurately handle the actual angle variations, such as rotated or tilted license plates, estimates of the angle of multi-directions may be required [23].
- Although the proposed algorithm efficiently handles non-uniform illuminations in the PKU dataset, when we test our algorithm on real life images that contain severe dark contrast at different day times and in unconstrained environments [29], a considerable decrease in detection accuracy is observed, as shown in Figure 5a. In such situations, we therefore recommend to use a state-of-the-art image enhancement algorithm to rectify the non-uniform illuminated images [28]. However, our proposed license plate detection algorithm accurately handles certain challenging scenes, for instance, extreme reflected glare as shown in Figure 3b.
- Color variation is an important aspect to be considered during the license plates study. In the PKU dataset, we have successfully detected different color license plates, such as Blue, Yellow, White, and Black, as shown in Figure 5a. We observe that when the vehicle and its license plate have the same color, the proposed algorithm might not yield encouraging results. Moreover, to detect vehicles and to locate license plates when RGB images are used, the HSV image is processed. We empirically observe that either RGB or HSV alone are insufficient to handle the sharp variations that occur in the input images.
- From the total of 3977 images containing 4263 license plates in the PKU database, the proposed algorithm accurately observed the license plates in 3939 images. As shown in Table 3, our proposed algorithm yields an average detection accuracy of 99.04% on this database. Our simulations also reveal that PKU-G5 category still needs considerable attention, on which the proposed algorithm yields 97.30% detection accuracy.
- We believe that the proposed work is an encouraging solution in the real-time detection of targeted license plates. After the successful detection, later, a suitable action can be taken, such as plate area estimation and recognition, vehicle owner identity, and theft/occlusion cases [30,31,32,33,34]. Similarly, for security applications, any suspicious object/plate can be tracked [35] and estimated [36,37].
- Since, the ALPD is a preliminary step for a license plate recognition system [38,39,40,41], any license plate recognition system is therefore highly dependent upon accurate and precise license plate detection that will be fed to the recognition system.

## 5. Conclusions

We discussed an effective license plate detection system in this study that used a smart integration of Faster R-CNN with image processing methods. To shorten the time it takes to detect the license plates, the suggested method first uses the Faster R-CNN to detect diverse vehicles in images. The detected vehicle image is next analyzed by the License Plate Localization Module (LPLM), which searches for the presence of license plate(s). For the PKU-G1–G5 categories, including extreme reflected glare situations, the LPLM analyzes the detected vehicle image, turns it into an HSV, applies morphological processes, and ultimately performs an aspect ratio test to spot the license plate(s). Extensive testing on difficult PKU vehicle datasets demonstrates the developed method’s resilience in comparison to prior approaches in plate(s) detection accuracy, precision, recall, and execution time. Moreover, in the PKU dataset, modifications in crucial characteristics, such as image resolution, illuminations, vehicle shapes, license plate angle, and color variations, had the least impact on the proposed technique.

In the future, important questions, for instance, partially occluded or broken license plates, dirty with soil, or large angle variation, can be further inspected. Once the license plates are accurately detected, a robust recognition algorithm can also be used to process the detected plates. Since, the developed method processes both the RGB and HSV color components, it can also be made capable to process only one-color domain images. Moreover, the proposed method can also be utilized in computationally low video frames. Finally, for real-time security applications, a parallel processing scheme of the proposed method can also be developed.

## Figures and Tables

**Table 1:** Acronyms and their explanation.

Acronym	Meaning
AdaBoost	Adaptive Boosting
ALPD	Automatic License Plate Detection
AR/CR	Aspect Ratio/Connected Region
(ψ)	Convolutional Feature Map
DCNN	Deep Convolutional Neural Networks
(⊕)/(·)	Dialation/closing operation
FP/FN/TP	False Positive/False Negative/True Positive
(Γ)	Feature Vector
(*t*)	Ground Truth
HOG	Histogram of Oriented Gradients
HSI/HSV	Hue Saturation Intensity/Value
ITS	Intelligent Transportation Systems
LPLM	License Plate Localization Module
(ξ)	Matching Confidence
PCA	Principal Component Analysis
PKU	Peeking University Dataset
RGB	Red, Green, and Blue
RoI	Region of Interest
RPN	Region Proposal Network
SA	Spatial Area

**Table 2:** Description of the PKU dataset.

Cat.	Conditions	Input Image Res. (Pixels)	No. of Images	No. of Plates	Plate Height (Pixels)
G1	Cars on roads; ordinary environment at different daytimes; contains only one license plate per image	1082 × 728	810	810	35–57
G2	Cars/trucks on main roads at different daytimes with sunshine; only one license plate in each image	1082 × 728	700	700	30–62
G3	Cars/trucks on highways during night; one license plate per image	1082 × 728	743	743	29–53
G4	Cars/trucks on main roads; daytimes with reflective glare; one license plate in input images	1600 × 1236	572	572	30–58
G5	Cars/trucks at roads junctions with crosswalks with several plates per image	1600 × 1200	1152	1438	20–60
	**The complete PKU dataset**	**Mentioned Above**	**3977**	**4263**	**20∼62**

**Table 3:** Vehicle detection accuracy.

Cat.	No. Vehicles	Resolution (Pixels)	Detection Accuracy %	Remarks
G1	810	600 × 340	100	Vehicle detection module yields detection accuracy over 99% for up to 3 vehicles appearing in an image. It successfully locates vehicles in frontal/side views. Moreover, it accurately spots vehicles in reflective glare.
G2	700	400 × 300	100	
G3	743	420 × 280	100	
G4	572	400 × 320	99.7	
G5	1438	300 × 270	99.1	
**Average**			**99.76**	

**Table 4:** License plate detection accuracy (%) comparison.

Ref	G1	G2	G3	G4	G5	Average
[21]	98.76	98.42	97.72	96.23	97.32	96.62
[22]	98.89	98.42	95.83	81.17	83.31	91.09
[23]	97.39	97.30	97.45	97.38	97.38	97.38
[24]	95.43	97.85	94.21	81.23	82.37	91.09
[25]	82.90	83.30	87.11	83.71	83.81	84.16
**Proposed**	**99.81**	**99.50**	**99.20**	**99.40**	**97.30**	**99.04**

## Data Availability

Data can be provided on demand.
